# There is poor accuracy in documenting the location of labral and chondral lesions observed during hip arthroscopy

**DOI:** 10.1186/s40634-020-0221-5

**Published:** 2020-02-01

**Authors:** Sanaz Hariri, Kyle R. Sochacki, Alex S. Harris, Marc R. Safran

**Affiliations:** 1 Stanford University, 450 Broadway St., M/C 6342, Redwood City, CA 94063 USA; 2 VA Palo Alto Health Care System, Bone and Joint Rehabilitation Center, 795 Willow Road (MC152), Menlo Park, CA 94025 USA

**Keywords:** Hip arthroscopy, Localization, Labrum, Femoral head, Acetabulum, Interobserver reliability, Accuracy

## Abstract

**Purpose:**

To determine and compare the accuracy and interobserver reliability of the different methods for localizing acetabular labral, acetabular chondral, and femoral head chondral lesions with hip arthroscopy .

**Methods:**

Three cadaver hips were placed in the supine position. Three labral, three femoral chondral, and six acetabular chondral lesions were made in each cadaver using electrocautery. Six surgeons classified the lesions according to different classification systems (clock-face, geographic, Method-G) using hip arthroscopy and standardized portals. Identification of each lesion was performed after conclusion of the study through open dissection and surgical hip dislocation to be used as the “gold-standard.” Accuracy was calculated as the number of correct answers divided by total number of responses for a given system. The interobserver reliability was calculated using the kappa coefficient. The different classification methods were compared. All *P* values were reported with significance set at *P* < 0.05.

**Results:**

The clock-face method had an accuracy of 74% (95% CI, 60%–85%) and interobserver reliability of 0.19 (95% CI, 0.11–0.26) while the geographic method had an accuracy of 50% (95% CI, 36%–64%) and interobserver reliability of 0.21 (95% CI, 0.05–0.31) for acetabular labral lesion identification (*P* > 0.05). The acetabular chondral lesion identification accuracy was 56% (95% CI, 46%–65%) for Method G, 66% (95% CI, 56%–75%) for Method G-simp, and 63% (95% CI, 53%–72%) for the geographic system (*P* > 0.05) with an interobserver reliability of 0.31 (95% CI, 0.27–0.35), 0.34 (95% CI, 0.28–0.40), and 0.40 (95% CI, 0.34–0.45), respectively (*P* > 0.05). Femoral chondral lesion identification accuracy was 74% (95% CI, 60%–85%) for Method G, 43% (95% CI, 29%–57%) for the geographic method, and 59% (95% CI, 45%–72%) for the geographic-simp system with interobserver reliability of 0.37 (95% CI, 0.27–0.47), 0.34 (95% CI, 0.28–0.40), and 0.40 (95% CI, 0.29–0.51), respectively (*P* > 0.05). Method G was significantly more accurate than the geographic system (*P* = 0.001).

**Conclusions:**

There was poor to fair accuracy and interobserver reliability of the reporting systems for localization of labral, acetabular chondral, and femoral chondral lesions encountered during hip arthroscopy. The study suggests there is a need for a new method that is easy to use, reliable, reproducible and accurate.

## Background

Hip arthroscopy is a common technique used to treat several conditions such as Femoroacetabular Impingement (FAI) syndrome, acetabular labral tears, chondral defects, hip instability, and extra-articular causes of hip pain [1–6]. Expanded indications and advancements in arthroscopic techniques have led to a substantial increase in hip arthroscopy utilization throughout the world [7–10]. The relatively late attention to hip arthroscopy, particularly when compared to arthroscopy of the shoulder and knee, can largely be attributed to the difficulty of evaluating the hip for pathology and the unique challenges of accessing and navigating the hip joint.

Burman has been widely credited for being the first to describe hip arthroscopy. However, in his landmark 1931 cadaver study describing this procedure, he only described arthroscopy of the peripheral compartment as he could not enter the central compartment of the hip joint [11]. The anatomy of the hip joint itself makes access to the joint and navigation within the joint more difficult than other joints. The femoral head is recessed within a deep concave acetabulum, creating considerable constraint. Thick muscles, ligaments, and capsule surround the joint, making distraction of the hip joint, penetration of the capsule, and maneuvering within the joint difficult.

Beck et al. provided a descriptive classification of the types of lesion in the hip, but there is little consensus on the best method for describing the location on the femoral head or acetabulum [12]. As techniques for hip arthroscopy continue to develop, it is important that clinicians be able to accurately describe the location of labral and chondral lesions for documentation, communication, and outcomes studies. Results of studies cannot be compared validly nor can multicenter trials be conducted effectively until there are accurate lesion localization systems with high interobserver reliability and consensus use.

The purpose of this study was to determine and compare the accuracy and interobserver reliability of the different methods for localizing acetabular labral, acetabular chondral, and femoral head chondral lesions with hip arthroscopy in the supine position. The authors hypothesized that there would be poor accuracy and interobserver reliability with all methods of identification and that there would be no significant difference between identification methods.

## Methods

Six high volume (range: 60–350 hip arthroscopies per year) hip arthroscopy surgeons (study surgeons) were recruited to participate in the study. The principle investigator and surgeons who had developed localization systems were excluded from the study surgeon group. All surgeons involved in the study routinely use the supine position for their hip arthroscopy. Three cadaver hips (two right and one left) were placed in the supine position in the lab. The three proctor surgeons established the viewing and working portals using fluoroscopic guidance for each cadaver. The proctor surgeons then created three labral, three femoral chondral, and six acetabular chondral lesions in their respective cadavers using electrocautery, based on a power analysis of the number of lesions required.

The study surgeons were presented the lesion localization systems utilized in the study and provided with a scoring sheet for each cadaver. The proctor surgeons were stationed at their respective cadavers and remained there for the full duration of the study. Each study surgeon identified the correct lesion during diagnostic arthroscopy and scribed the location of the lesions using the different methods on the scoring sheet. The proctor surgeons ensured that each lesion was localized and classified using each system.

An open dissection and surgical hip dislocation was performed for each cadaver at the conclusion of the study. Under direct visualization, the principle investigator recorded the true anatomic location of the lesions to be used as the “gold-standard.”

**Fig. 1:**
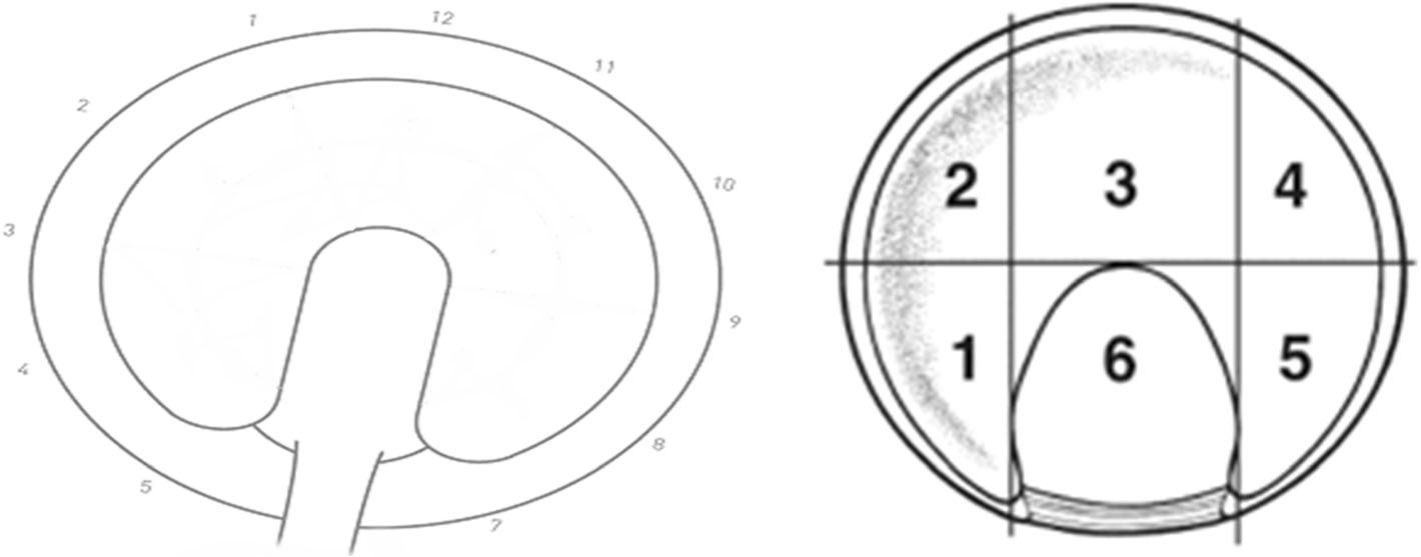
Acetabular Labral Lesion Classification Systems. The right hip zones for the clock-face (left) and geographic (right) methods. Reprinted from Arthroscopy, 24 (5), Ilizaliturri, V. M. et al., A geographic zone method to describe intra-articular pathology in hip arthroscopy: cadaveric study and preliminary report, 534–539, 2007, with permission from Elsevier

**Fig. 2:**
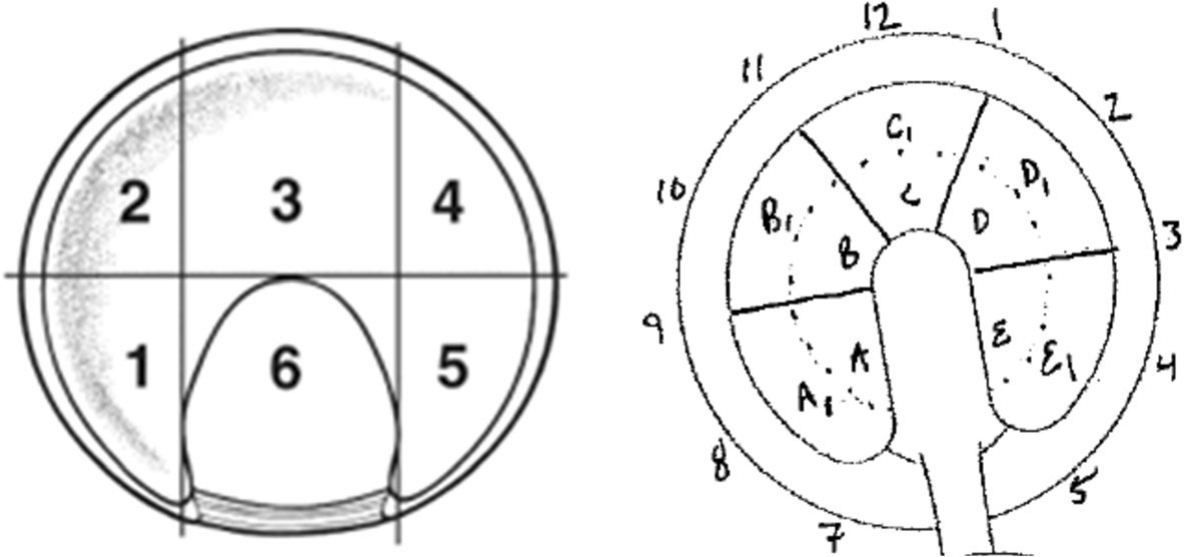
Acetabular Chondral Lesion Classification Systems. The right hip zones for the geographic (left) and Method G (right) methods. Reprinted from Arthroscopy, 24 (5), Ilizaliturri, V. M. et al., A geographic zone method to describe intra-articular pathology in hip arthroscopy: cadaveric study and preliminary report, 534–539, 2007, with permission from Elsevier

**Fig. 3:**
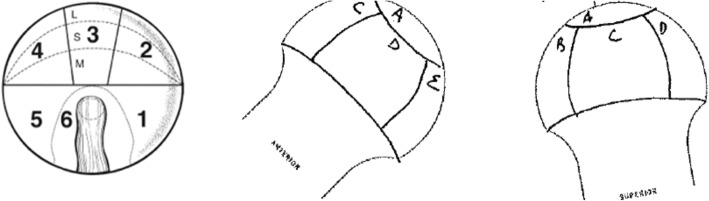
Femoral Head Chondral Lesion Classification Systems. The right hip zones for the geographic (left) and Method G (right) methods. Reprinted from Arthroscopy, 24 (5), Ilizaliturri, V. M. et al., A geographic zone method to describe intra-articular pathology in hip arthroscopy: cadaveric study and preliminary report, 534–539, 2007, with permission from Elsevier

The clock-face and geographic methods were utilized for labral lesion (Fig. 1) location classification [13, 14]. The methods described by Griffin et al. (Method G) and the geographic system were used for acetabular (Fig. 2) and femoral (Fig. 3) chondral lesions [13, 15]. Additionally, a simplified geographic method (geographic-simp) was used for femoral chondral lesions. The distinction between medial (M), superior (S), and lateral (L) zones was removed (e.g. 2 M, 2S, and 2 L were treated as one zone, such that the femoral head location did not include medial, superior, and lateral sub-regions). The simplified Method G (Method G-simp) system was also used for analysis of the acetabular chondral lesions. Under this system, the distinction between subzones Letter and Letter1 was removed (e.g. A and A1 were treated as one zone, removing the distinction between central and peripheral lesions on the acetabulum).

### Statistics

Statistical analysis was performed using STATA version 10. A priori power analysis was performed and it was determined that 12 lesions per cadaver, with 3 cadavers and 6 surgeons, would be adequate for a power of 80% and *P* < .05. Accuracy was calculated as the number of correct answers divided by the total number of responses for a given system. The correct answer was defined as the location of the lesion identified after the arthrotomy and surgical dislocation of the hip (gold-standard). The interobserver reliability was calculated using the kappa coefficient with values ≤0 indicating no agreement, 0.01–0.20 as poor, 0.21–0.40 as fair, 0.41–0.60 as moderate, 0.61–0.80 as substantial, and 0.81–1.00 as almost perfect agreement [16]. Accuracy and kappa coefficients for each method were reported with respective 95% confidence intervals (CI). All *P* values were reported with significance set at *P* < 0.05.

## Results

**Fig. 4:**
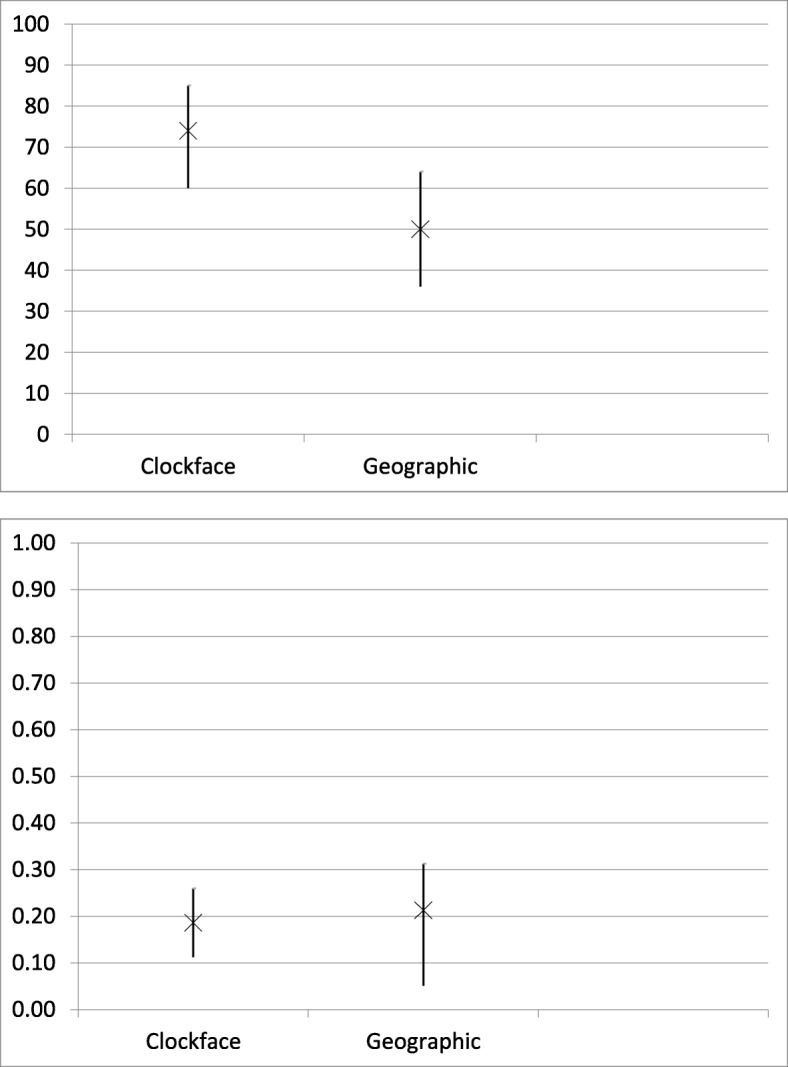
Acetabular Labral Lesion Accuracy (top) and Interobserver reliability (bottom). X = accuracy (%) or kappa coefficient with 95% confidence interval

The accuracy for acetabular labral lesion identification was 74% (95% CI, 60%–85%) for the clock-face system and 50% (95% CI, 36%–64%) for the geographic method (*P* > 0.05). The interobserver reliability for labral lesion identification was 0.19 (95% CI, 0.11–0.26) for the clock-face system and 0.21 (95% CI, 0.05–0.31) for the geographic method (*P* > 0.05) (Fig. 4).

**Fig. 5:**
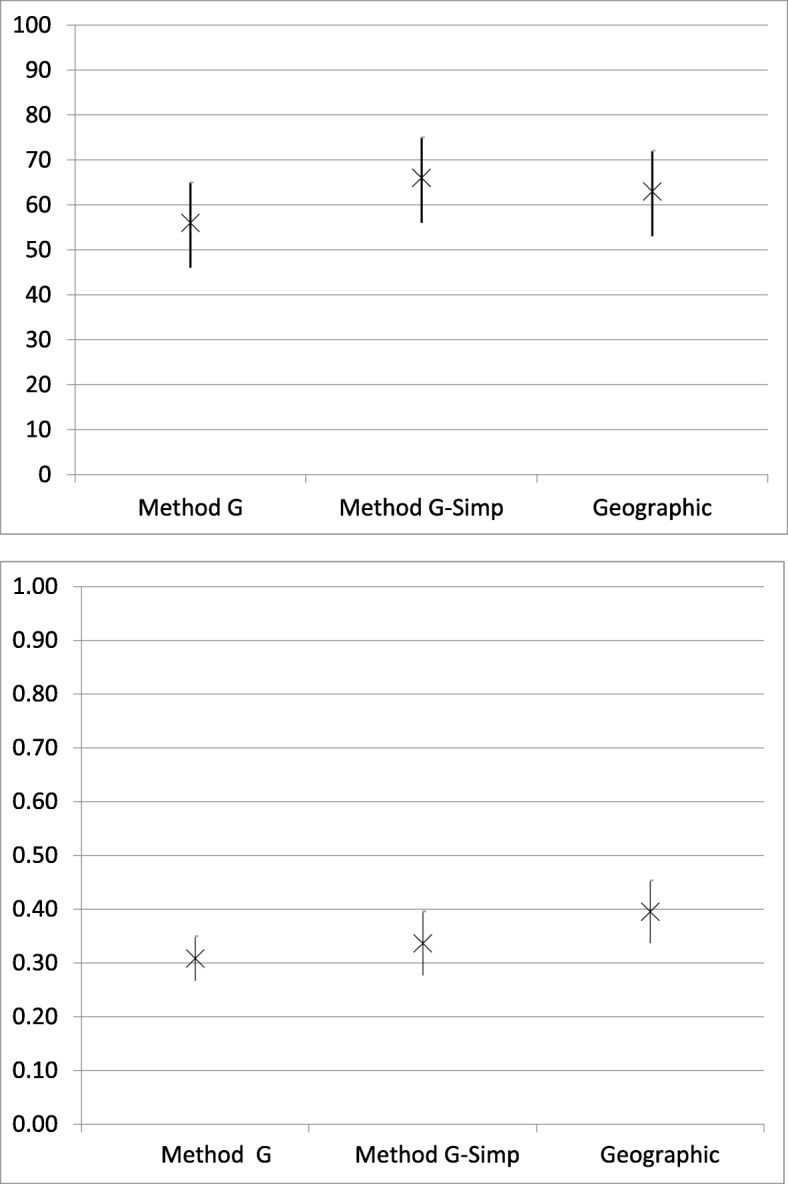
Acetabular Chondral Lesion Accuracy (top) and Interobserver reliability (bottom). X = accuracy (%) or kappa coefficient with 95% confidence interval

The acetabular chondral lesion identification accuracy was 56% (95% CI, 46%–65%) for Method G, 66% (95% CI, 56%–75%) for Method G-simp, and 63% (95% CI, 53%–72%) for the geographic system (*P* > 0.05). The interobserver reliability for acetabular chondral lesion identification was 0.31 (95% CI, 0.27–0.35) for Method G, 0.34 (95% CI, 0.28–0.40) for Method G-simp, and 0.40 (95% CI, 0.34–0.45) for the geographic system (*P* > 0.05) (Fig. 5).

**Fig. 6:**
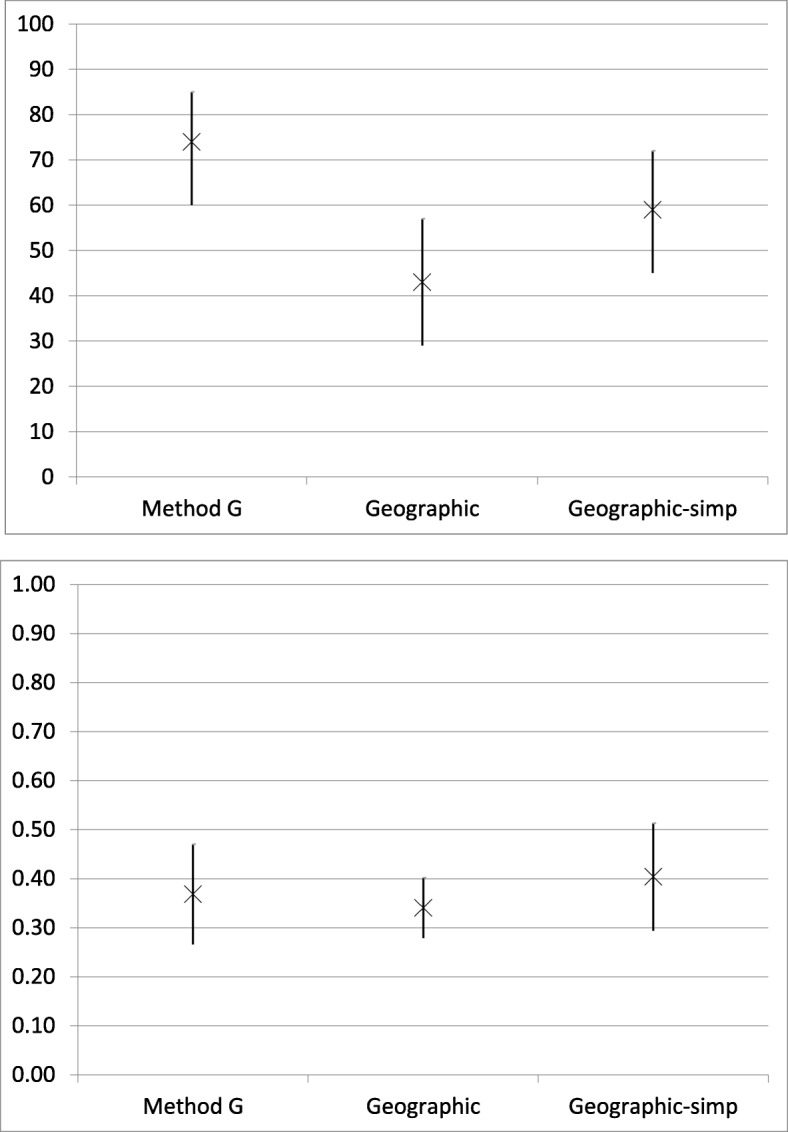
Femoral Chondral Lesion Accuracy (top) and Interobserver reliability (bottom). X = accuracy (%) or kappa coefficient with 95% confidence interval

Femoral chondral lesion identification accuracy was 74% (95% CI, 60%–85%) for Method G, 43% (95% CI, 29%–57%) for the geographic method, and 59% (95% CI, 45%–72%) for the geographic-simp system. Method G was significantly more accurate than the geographic system (*P* = 0.001) but there was no significant difference compared to the geographic-simp system for femoral chondral lesion identification (*P* > 0.05). There was also no significant difference between the accuracy of the geographic and the geographic-simp systems for femoral chondral lesion identification (*P* > 0.05). The interobserver reliability for femoral chondral lesion identification was 0.37 (95% CI, 0.27–0.47) for Method G, 0.34 (95% CI, 0.28–0.40) for the geographic method, and 0.40 (95% CI, 0.29–0.51) for the geographic-simp system (*P* > 0.05) (Fig. 6).

## Discussion

It was determined that the accuracy of the different methods of localizing acetabular labral, acetabular chondral, and femoral head chondral lesions with hip arthroscopy ranged from 43% to 74% with interobserver reliability that was poor to fair. Additionally, Method G was significantly more accurate than the geographic system, but no other significant differences in accuracy or interobserver reliability were observed between classification systems. This study partially confirmed the authors’ hypotheses.

The Beck and Outerbridge systems are the most commonly used for classifying the type of acetabular or femoral lesion in hip arthroscopy [12, 17, 18]. However, there is no consensus on the best method of describing the location of the lesion. Without such a system, comparing the results of different studies remains limited. This is especially important as lesion location has been determined to have prognostic implications [19].

The clock-face method is the traditional and most commonly used method for identifying chondral and labral lesions of the acetabulum [14]. Although easy to understand, there are several problems associated with this method of localization. The 12 o’clock position is at the most lateral and superior aspect of the acetabulum, and the 6 o’clock position is located at the middle of the transverse ligament. However, due to the unique anatomy of the hip, the transverse ligament is not the most inferior point of the acetabulum and can be difficult to visualize with arthroscopy. Additionally, the anterior and posterior aspects of the acetabulum lie either between 1 and 5 o’clock or 7 and 11 o’clock depending on the laterality of the hip. This may be confusing for some surgeons as the perception of these positions may be altered arthroscopically based on acetabular version and inclination.

As such, Ilizaliturri et al., introduced the geographic method of acetabular and femoral head localization [13]. This method divided the acetabulum and femoral head into 6 zones that were independent of laterality. Using 6 surgeons observing a surgeon performing arthroscopy on one cadaver, the authors found this method to be more reproducible than the clock-face system for localizing acetabular and femoral head lesions [13]. This differs compared to the current study where the geographic system was found to 50% accurate for identifying acetabular labral lesions (interobserver reliability of 0.21), 63% accurate for acetabular chondral lesions (interobserver reliability of 0.40), and 43% accurate for femoral chondral lesions (interobserver reliability of 0.34). The geographic method was significantly less accurate compared to the Method G for femoral chondral lesions and no significant difference was found between the geographic system and clock-face method.

One potential reason for this difference may be due to their use of inter-item correlations compared to the accuracy and interobserver reliability used in the present study. While inter-item correlations provide information about the internal consistency of the classification system itself, it does not allow one to determine if the method is able to be reliably used between different surgeons. Additionally, the study by Ilizaliturri et al. did not compare their system to a gold standard through open dissection and the surgeons included in their study contributed to the development of their geographic zone method leading to potential bias and confounding.

The Method G system is a combination of the clock-face and geographic methods for localizing chondral lesions on the acetabulum and femoral head [15]. Due to the familiarity with these methods, it is not surprising that when used in the current study, the Method G or Method G-simple were the most accurate (66% for acetabulum and 74% for femoral head). This reached significance when comparing Method G to the geographic method on the femoral head (*P* = 0.001). The improved accuracy is likely due to Method G only having 5 possible zones on the femoral head compared to 6 (geographic-simple) or 12 (geographic). As such, with fewer potential zones, there is less chance for making an incorrect selection, but the localization is less precise.

Despite the improved accuracy, however, this same trend was not seen with interobserver reliability where no method was shown to be superior. The interobserver reliability ranged from 0.19 (clock-face method for the acetabular labrum) to 0.40 (geographic system and geographic-simple method for chondral lesions of the acetabulum and femur, respectively) in the present study with seven localization methods determined to be fair and one poor. This illustrates the need for the development or universal adoption of a localization system that is both accurate and can be reliably used by hip arthroscopists all over the world.

The low accuracy and interobserver reliability of the localization methods used in the present study are particularly discouraging as we would expect the hip arthroscopy experts to be the best at identifying and localizing lesions. If high volume hip arthroscopy surgeons are unable to accurately and reliably classify lesions, how can one expect novice or lower-volume hip arthroscopy surgeons to do so? It is therefore perhaps not meaningful to report the location of chondral and labral lesions or attempt to correlate the location with prognosis at this time. In order to improve reliability and accuracy of reporting systems, the authors recommend standardizing a single method and teaching that method during training, courses, and conferences going forward.

There are some limitations to this study. There were only six surgeons and 3 cadavers utilized in the present study leading to the potential for selection bias. Despite these low numbers, a prior power analysis determined the sample size to be sufficient and adequately powered. The results of the study may not be extrapolated to all surgeons with differing skill levels due to the extensive hip arthroscopy experience of the surgeons included in the study. However, it is likely that novice hip arthroscopists would have fared even worse. Additionally, not all surgeons used the same cadavers. This may have led to differences in specimen quality, visualization, and left-right paradox of the clock-face experienced by the different surgeons.

## Conclusions

There was poor to fair accuracy and interobserver reliability of the reporting systems for localization of labral, acetabular chondral, and femoral chondral lesions encountered during hip arthroscopy. The study necessitates the need for a new method that is easy to use, reliable, reproducible and accurate.
